# No correlation to collagen synthesis disorders in patients with Perthes’ disease: a nationwide Swedish register study of 3433 patients

**DOI:** 10.1186/s12891-023-07161-8

**Published:** 2024-01-09

**Authors:** M Lindblad, M Bladh, H Björnsson-Hallgren, G Sydsjö, T Johansson

**Affiliations:** 1 Department of Emergency Medicine, Linköping University, Norrköping, Sweden; 2 Department of Biomedical and Clinical Sciences, Linköping University, Linköping, Sweden; 3 Department of Obstetrics and Gynaecology, Linköping University, Linköping, Sweden; 4 Department of Orthopaedics, Linköping University, Linköping, Sweden; 5 Department of Orthopaedics, Linköping University, Norrköping, Sweden

**Keywords:** Perthes’ disease, COL2A1, Collagen synthesis disorder, Osteonecrosis of the hip, Birth characteristics

## Abstract

**Background:**

Mutations of the COL2A1 gene have been identified in patients with Perthes’ disease. Several studies have hypothesised a connection between Perthes’ disease and collagen synthesis disorders, especially COL2A1-related disorders, but no large studies on the subject have been made. The aim of this study was thus to discover if there is a connection between patients presenting with Perthes’ disease, and collagen synthesis disorders. A secondary aim was to see if the children with both disorders had less optimal birth characteristics than the rest.

**Methods:**

Swedish national registers were used to collect data on children diagnosed with Perthes’ disease or a collagen synthesis disorder. These registers include all births in Sweden, and data from both outpatient and in-hospital visits. A wide range of data is included besides diagnoses. All children with follow-up data to the age of 15 years were included. Pearson’s chi-square was used for analysis. Statistical significance was further analysed with Fisher’s Exact Test.

**Results:**

In total, 3433 children with either diagnosis were included. 1620 children had only Perthes disease, while 1808 children had only a collagen synthesis disorder. Five children were found to have both the diagnosis Perthes’ disease and a collagen synthesis disorder. One child was large for their gestational age and none of the children had a low birthweight. Two of the children were moderately preterm.

**Conclusions:**

The distinct lack of overlap in such a large body of material raises doubt about a connection between the presentation of Perthes’ disease and collagen synthesis disorders, either COL2A1-related or not. We could not find an overrepresentation of less optimal birth characteristics either.

## Introduction

Perthes’ is a disease of the hip that presents in childhood with osteonecrosis of the femoral head. The incidence varies; a recent study on a Swedish population found an incidence of 9.3 per 100 000 subjects [1]. The cause for Perthes’ disease is unknown but it is most often described as multifactorial. One hypothesis is a disturbance in collagen type II synthesis and structure [2–5]. Collagen type II is the major structural component of the extracellular matrix in hyaline cartilage, intervertebral discs, retina, sclera and lenses of the eyes. More than 90 known mutations have been described with varying severity from mild symptoms to intrauterine death [6]. Dysplastic disorders affecting the musculoskeletal system have all been described related to different types of mutations in the COL2A1 gene, but they share characteristics [7]. Studies have also shown the presence of COL2A1 mutations in patients with Perthes disease. Crofton et al. found lower soft tissue collagen synthesis and enhanced collagen breakdown in children with Perthes’ disease compared to healthy controls [2]. Su et al. found a mutation of COL2A1 that caused abnormal chondrocytes, disarrangement of fibres and decreased stability in the femoral head cartilage of patients with Perthes’ disease [8]. Further support for mutations in the COL2A1 gene in patients with Perthes’ disease has been published by several authors [3–5, 9, 10].

Other collagen synthesis disorders also have an effect on the musculoskeletal system. Ehler-Danlos syndrome (EDS) has different subtypes with multiple different defects in the collagen synthesis system. In Osteogenesis Imperfecta (OI) the synthesis of type I collagen is decreased or defective [11]. (Table 1)

**Table 1:** Incidences of Perthes’ disease and collagen synthesis disorders in this study

Disorder	Incidence
Perthes’ disease	9.3 / 100 000
Spondyloepiphyseal dysplasia	1 / 100 000
Kniest dysplasia	0.1 /100 000
Stickler dysplasia	10–30 / 100 000
Czech dysplasia	No known incidence, 11 known families
Megaepiphyseal dysplasia	10 / 100 000
Oto-spondylo-megaepiphyseal dysplasia	No known incidence, 30 known cases
Akondrogenesis-hypochondrogenesis type II	1.6–2.5 / 100 000
Osteogenesis imperfecta	6–20 / 100 000
Ehler-Danlos syndrome	20 / 100 000

Incidences of collagen synthesis disorders have been compiled from the website for rare diseases by the Swedish National Board of Health and Welfare [12] and the European database for rare diseases Orphanet [13]

Due to the defect collagen found in patients with Perthes’ disease in these studies, a possible link between Perthes’ disease and other COL2A1-related disorders has been suggested [2–5, 8–10]. However, these studies are based on small populations and different mutations in the gene. In a case study of two patients with Perthes’ disease, Kannu et al. described some of these COL2A1-related disorders [9]. One older study raised the possibility of problems with cross-diagnosis between COL2A1-related disorders and Perthes’ disease, as opposed to comorbidities [14]. However, one case study found OI and Perthes’ disease in the same patient [15]. No larger studies exploring a possible connection between Perthes’ disease and other COL2A1-related disorders have been performed to our knowledge and no studies have investigated a connection with non-COL2A1 related collagen synthesis disorders.

Children with EDS and OI have an elevated risk for premature birth, low birthweight and a low crown-heel length [16], just like children with Perthes’ disease [17].

The primary aim was to study the correlation between Perthes’ disease and COL2A1-related disorders. The secondary aim was to explore any connection to non-COL2A1-related collagen synthesis disorders. The third aim was to study the birth characteristics of the children included.

## Methods

### Registers

Data was collected from several population-based national registers using the personal identification number assigned to every person residing in Sweden [18–23]. The first register used was the Medical Birth Register [20], which includes 97–99% of pregnancies that have resulted in births in Sweden since 1973. This register is based on medical charts from services providing maternal health care, obstetric care and infant care. Information on pregnancy, delivery and neonatal health was collected from this register. The second register used was the Swedish National Patient Register [23, 24], with information on age, sex and diagnoses on all patients discharged from hospital or treated in outpatient care since 1987. Data in these registers, including diagnoses, is registered by all medical doctors in Sweden irrespective of specialty. Children were identified as having been diagnosed with Perthes’ disease if they had the following diagnoses: ICD-8 722.11 and 722.19; ICD-9 732B; and ICD-10 M91.1, M91.2 and M91.3. These children served as cases; all other children were used as controls.

The COL2A1-related disorders included in this study were spondyloepiphyseal dysplasia (SED) including the congenital form and the form with premature onset arthrosis, Kniest dysplasia, Stickler dysplasia, Czech dysplasia, megaepiphyseal dysplasia (MED), oto-spondylo-megaepiphyseal dysplasia (OSMED) and akondrogenesis-hypochondrogenesis type II [3, 4, 8]. Children were identified as having been diagnosed with one of these if they had the following diagnoses: ICD-8 756.4; ICD-9 755.5, 756.0, 756.4, 756.5 and 759.8; and ICD-10 Q77.0, Q77.7 and Q87.0.

Non-COL2A1-related collagen synthesis disorders included in this study were OI and EDS. Children were identified as having been diagnosed with one of these if they had the following diagnoses: ICD-8 756.5; ICD-9 756.51 and 756.83; and ICD-10 Q78.0 and Q79.6.

To evaluate a connection to less optimal birth characteristics, information on the children’s birthweight, term and size in relation to gestational age was collected.

### Study population

**Fig. 1:**
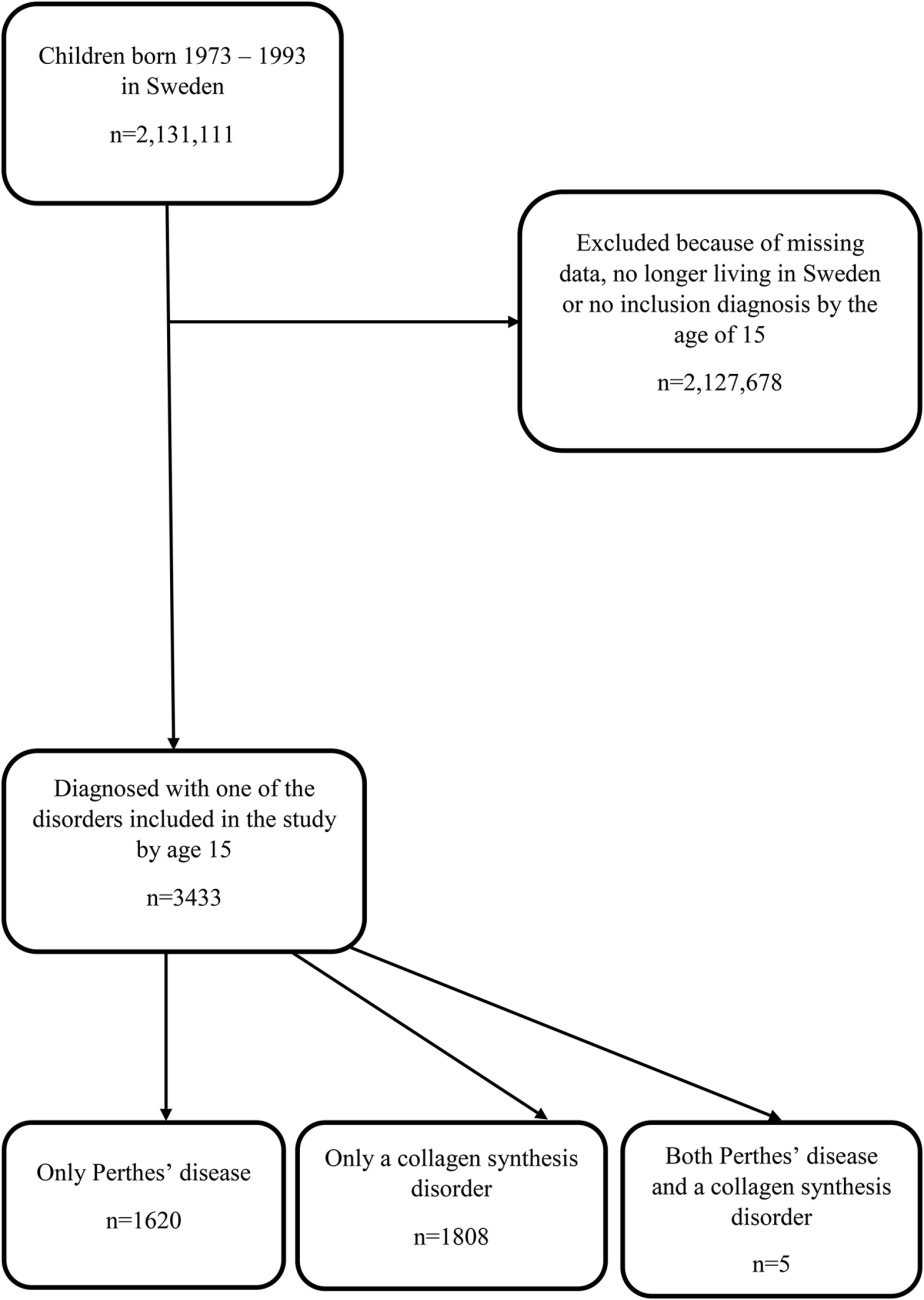
Inclusion process and selection of study groups

The study population was defined as all children born in the period 1973–1993 and living in Sweden at 15 years of age. The register data covers the years 1973–2004. The study population was chosen to make it possible to retrieve data on children within the cut-off age. The cut-off age of 15 was chosen based on the fact that all the children’s diagnoses included in this study debuted before this age. Individuals who were deceased before their 15th birthday or who did not reside in Sweden at the age of 15 were excluded. Individuals with missing data were excluded. (Fig. 1)

### Statistical methods

The univariate correlation between Perthes’ disease and collagen synthesis disorders was evaluated using Pearson’s chi-square test. Statistical significance was further analysed with Fisher’s Exact Test. All statistical analyses were performed using IBM SPSS, version 27 (IBM SPSS Inc., Armonk, NY). *P*-value < 0.05 was defined as statistical significance (two-sided).

## Results

In total, 3433 children had been diagnosed with either Perthes’ disease or a collagen disorder. Most of these children, 1620, had only Perthes’ disease, and 1808 children had a collagen disorder diagnosis. Five patients had both Perthes’ disease and a collagen disorder diagnosis. (Table 2)

**Table 2:** Distribution of collagen synthesis disorders and Perthes’ disease in the study population

	Perthes’ disease		
	Yes n (%)	No n (%)	*p*-value*
Collagen synthesis disorder			< 0.001
Yes	5 (0.1)	1808 (100.0)	
No	1620 (99.9)	0 (0.0)	

Of the five children who had both diagnoses, one had OI, three had SED and one had Stickler dysplasia. Two of the children were boys and three were girls. One child was large for gestational age. None of the children had a low birthweight. Two of the children were moderately preterm. (Table 3)

**Table 3:** Birth characteristics of children born with Perthes’ disease or a collagen synthesis disorder*

	Perthes’ disease n (%)	Collagen synthesis disorder n (%)
Size for gestational age		
Large for gestational age	87 (5.4)	151 (8.3)
Small for gestational age	50 (3.1)	37 (2.0)
Average for gestational age	1488 (91.6)	1625 (89.6)
Birthweight		
Very low birth weight, < 1,500 g	19 (1.2)	40 (2.2)
Low birth weight, 1,500-2,499 g	75 (4.6)	123 (6.8)
Normal birth weight, ≥ 2,500 g	1531 (94.2)	1650 (91.0)
Gestational age		
Very preterm, < 32 weeks	14 (0.9)	36 (2.0)
Moderately preterm 32–36 weeks	112 (6.9)	157 (8.7)
Term, 37–42 weeks	156 (9.6)	170 (9.4)
Post term, > 42 weeks	1343 (82.6)	1450 (80.0)

*Five children had been diagnosed with both Perthes’ disease and a collagen synthesis disorder and are included in both diseases

## Discussion

The main finding in this study was that there was no correlation either between Perthes’ disease and collagen synthesis disorders in general, or disorders specifically related to COL2A1 mutations. In fact, there was barely any overlap at all. Only five children had both Perthes’ disease and a collagen synthesis disorder.

The lack of overlap between the groups was very distinct, which strongly brings into question a correlation between Perthes’ disease and collagen synthesis disorders. As previously mentioned, some case reports have described children with both Perthes’ disease and COL2A1-related collagen synthesis disorders. It is possible that this combination is simply very rare. Also, some phases of Perthes’ disease can be difficult to differentiate from other necrotic or degenerative hip joint disorders. It is possible that the children in these case reports, and possibly even some of the overlapping cases in this study, have actually been misdiagnosed and do not in fact have Perthes’ disease. The changes in their hip joints could possibly be caused by the collagen synthesis disorder itself.

The birth characteristics included in this study did not show any significant results. The supposition was that the children with both diagnoses would have less optimal birth characteristics, but this was not the case. The children were mainly average size or even large with a normal birthweight, and none were very preterm.

This is, to our knowledge, the largest amount of patient data used for this type of study, which suggests that there is no strong correlation between Perthes and COL2A1-related collagen synthesis disorders. The study population includes most, if not all children in Sweden 1973–2004. It is possible that our study missed some children in either diagnosis group, but it is unlikely that this would be a significant number. Swedish national registers are very well maintained and comprehensive. It is unlikely that a child with one of these disorders would have gone unnoticed by the Swedish health care system. The manifestations of collagen synthesis disorders are quite visible and usually affect the child’s development and function markedly. They also debut quite early in childhood, and Swedish children are followed by child welfare centres for several years, facilitating early diagnosis. Perthes’ disease does not produce such visible changes, but causes pain and limping that are unlikely to go unnoticed by parents or health care providers.

The main limitation of this study is that we do not know if the children included actually had a genetic abnormality. Nowadays it is standard to conduct genetic testing on children suspected of having a collagen synthesis disorder, but it is uncertain how long this has been the case and we have no way of finding out if the children were actually tested. Furthermore, the diagnosis of Perthes’ disease is based on clinical presentation and radiological findings. These children never undergo any genetic testing as part of the diagnosis process. Therefore, this study should be viewed as a way of finding out if a strong correlation was suggested, and as an aid in determining whether more specific studies comparing patients with the same mutation but different presentations are needed.

## Conclusions

The results of this study imply that although Perthes’ disease could be at least partially caused by mutations in the COL2A1 gene, it appears to be a separate disorder from the other, more closely related COL2A1-associated disorders. It would be interesting to further map the different mutations in the COL2A1 gene, and see if certain types of mutations are exclusive to Perthes’ disease. Exclusive mutations could lead to the hypothesis of a stronger genetic aetiology. If the mutations are the same but the presentations have no correlation, a hypothesis could be made that some environmental factors or a combination of several genetic mutations are required for a specific presentation.

## Data Availability

The data that support the findings of this study are available from the Swedish National Board of Health and Welfare but restrictions apply to the availability of these data, which were used under licence for the current study, and so are not publicly available. Anyone interested in viewing the data needs to file an application with the Swedish National Board of Health and Welfare.

## Abbreviations

SED: Spondyloepiphyseal dysplasia
MED: Megaepiphyseal dysplasia
OSMED: Oto-spondylo-megaepiphyseal dysplasia
EDS: Ehler-Danlos syndrome
OI: Osteogenesis imperfecta
LGA: Large for gestational age
SGA: Small for gestational age
AGA: Average for gestational age
VLBW: Very low birthweight
LBW: Low birthweight
VPT: Very preterm
PT: Preterm
