# Correction: No correlation to collagen synthesis disorders in patients with Perthes’ disease: a nationwide Swedish register study of 3433 patients

**DOI:** 10.1186/s12891-025-08907-2

**Published:** 2025-07-25

**Authors:** M Lindblad, M Bladh, H Björnsson-Hallgren, G Sydsjö, T Johansson

**Affiliations:** 1 Department of Emergency Medicine, Linköping University, Norrköping, Sweden; 2 Department of Biomedical and Clinical Sciences, Linköping University, Linköping, Sweden; 3 Department of Obstetrics and Gynaecology, Linköping University, Linköping, Sweden; 4 Department of Orthopaedics, Linköping University, Linköping, Sweden; 5 Department of Orthopaedics, Linköping University, Norrköping, Sweden

**Correction: BMC Musculoskelet Disord 25, 42 (2024)**

**https://doi.org/10.1186/s12891-023-07161-8**

Following the publication of the original article [1], there was unfortunately a typing mistake in the published article mixing the numbers 3 and 8 in some places. The total of patients with at least one of the diagnoses is 3433 and not 3488. The subheading of the title reads “a nationwide Swedish register study of 3488 patients.” The correct subheading should be “a nationwide Swedish register study of 3433 patients.” In the Result section of the abstract, the sentence “In total, 3488 children with either diagnoses were included” is incorrect. The correct sentence is “In total, 3433 children with either diagnoses were included”. In the Results section of the article the first sentence of the first paragraph “In total, 3488 children had been diagnosed with either Perthes’ disease or a collagen disorder” is incorrect. The correct sentence should be “In total, 3433 children had been diagnosed with either Perthes’ disease or a collagen disorder”. The number of patients with a collagen synthesis disorder is 1808 and not 1803 as written in Table 2. The table with corrected numbers has been included below. In Figure 1, the box with results for both diagnosis groups is duplicated, and the box for patients with only Perthes’ disease is missing. In Figure 1 the number of excluded cases is incorrect, because of later adjustments in the calculations. The box for exclusions should be 2 127 678 and not 2 127 623. The flow chart with the correct numbers has been included below.

**Table 2:** Distribution of collagen synthesis disorders and Perthes’ disease in the study population

**Perthes’ disease**			
	**Yes**	**No**	*p*-value*
	**n (%)**	**n (%)**	
Collagen synthesis disorder			< 0.001
Yes	5 (0.1)	1808 (100.0)	
No	1620 (99.9)	0 (0.0)	

The corrected Fig. 1:

**Fig. 1:**
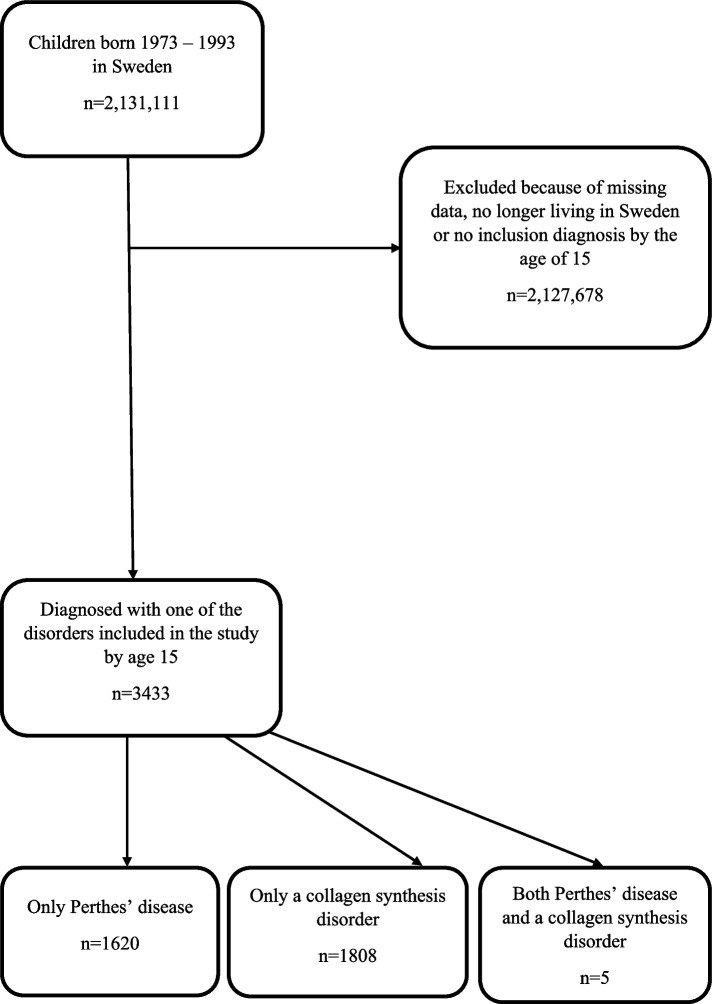
Inclusion process and selection of study groups

The old version of Fig. 1:

**Fig. 1:**
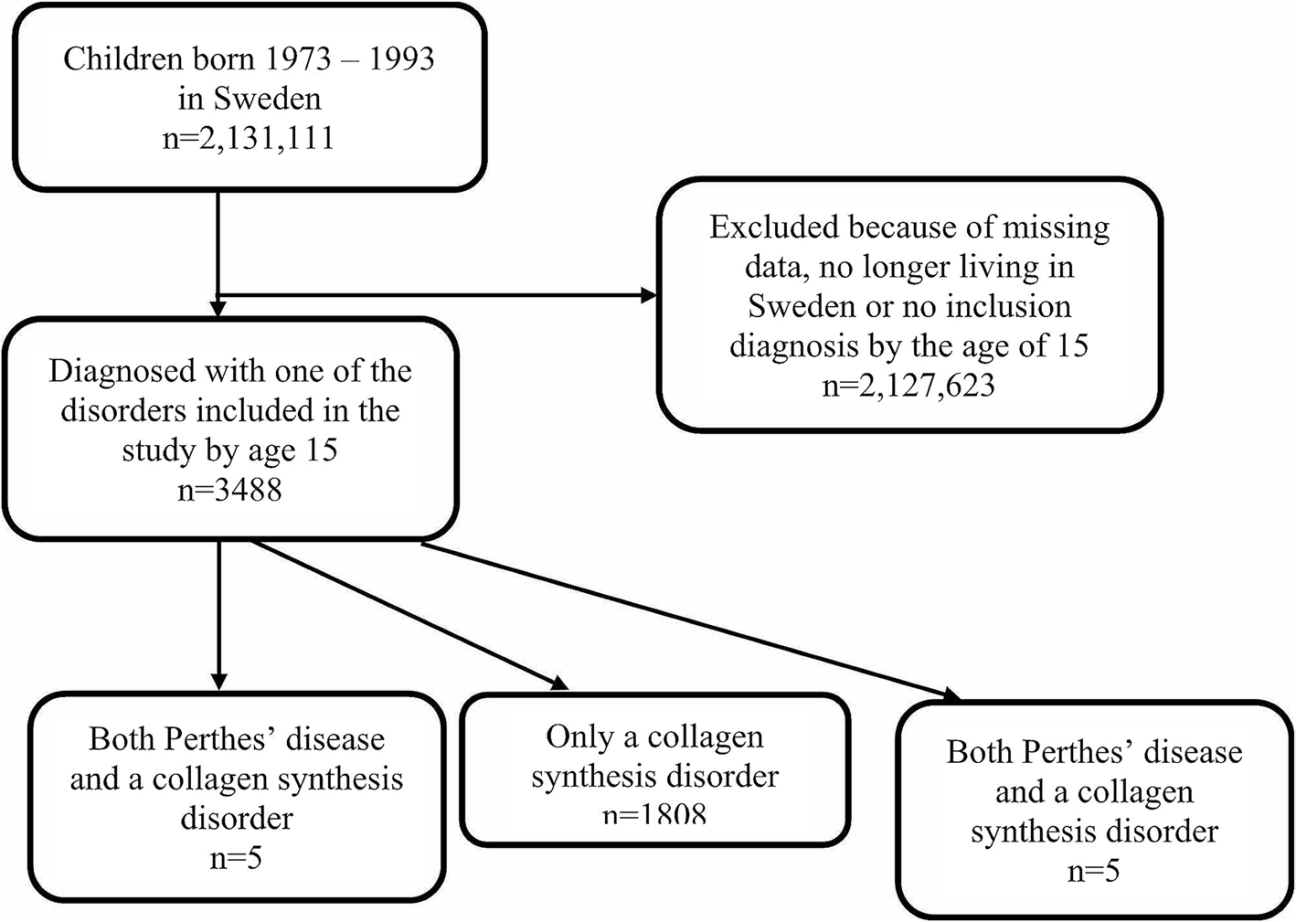
Inclusion process and selection of study groups
